# Assessment of the quality of primary care for the elderly according to the Chronic Care Model^1^

**DOI:** 10.1590/1518-8345.2331.2987

**Published:** 2018-03-08

**Authors:** Líliam Barbosa Silva, Sônia Maria Soares, Patrícia Aparecida Barbosa Silva, Joseph Fabiano Guimarães Santos, Lívia Carvalho Viana Miranda, Raquel Melgaço Santos

**Affiliations:** 2 Doctoral student, Escola de Enfermagem, Universidade Federal de Minas Gerais, Belo Horizonte, MG, Brazil. Scholarship holder at Coordenação de Aperfeiçoamento Pessoal de Nível Superior (CAPES), Brazil.; 3 PhD, Associated Professor, Escola de Enfermagem, Universidade Federal de Minas Gerais, Belo Horizonte, MG, Brazil.; 4 PhD.; 5 PhD, Physician, Hospital Governador Israel Pinheiro, Belo Horizonte, MG, Brazil.; 6 MSc, RN, Centro de Saúde, Secretaria Municipal de Saúde, Belo Horizonte, MG, Brazil.; 7 Undergraduate student in Nursing, Escola de Enfermagem, Universidade Federal de Minas Gerais, Belo Horizonte, MG, Brazil.

**Keywords:** Health of the Elderly, Primary Health Care, Self Care, Outcome and Process Assessment (Health Care), Diabetes Mellitus, Hypertension

## Abstract

**Objective:**

to evaluate the quality of care provided to older people with diabetes mellitus and/or hypertension in the Primary Health Care (PHC) according to the Chronic Care Model (CCM) and identify associations with care outcomes.

**Method:**

cross-sectional study involving 105 older people with diabetes mellitus and/or hypertension. The Patient Assessment of Chronic Illness Care (PACIC) questionnaire was used to evaluate the quality of care. The total score was compared with care outcomes that included biochemical parameters, body mass index, pressure levels and quality of life. Data analysis was based on descriptive statistics and multiple logistic regression.

**Results:**

there was a predominance of females and a median age of 72 years. The median PACIC score was 1.55 (IQ 1.30-2.20). Among the PACIC dimensions, the “delivery system design/decision support” was the one that presented the best result. There was no statistical difference between the medians of the overall PACIC score and individual care outcomes. However, when the quality of life and health satisfaction were simultaneously evaluated, a statistical difference between the medians was observed.

**Conclusion:**

the low PACIC scores found indicate that chronic care according to the CCM in the PHC seems still to fall short of its assumptions.

## Introduction

The progressive aging of the population has led to an increase in chronic conditions, especially diabetes *mellitus* and hypertension, the main primary causes of cardiovascular diseases on the world stage. These diseases were responsible for 14.4 million deaths in 2015 and 81.6 million years lost as disability-adjusted life years (DALY) worldwide^1^. Failures in the management of these chronic conditions contribute to negative impacts on the health of the population, with more late complications, rehospitalizations and lower quality of life, as well as an economic burden on health systems and family structure^2^. 

Given this scenario, the magnitude of problems associated with aging will depend a great deal on how healthy, or sick, or dependent on others, people will be in the extra years of life. This represents challenges to be overcome by the health sector in the search for an active and healthy aging^3^, mainly in the area of ​​Primary Health Care (PHC), considered a privileged *locus* for operationalization of actions aimed at health promotion and disease prevention.

In order to maintain the functionality of the elderly, it is essential to optimize the management of chronic conditions. This requires multidimensional strategies anchored in the concept of health conditions and with theoretical references related to patient-centered care^4^ ^-^ ^5^, which is not always observed. Studies have shown a persistent care practice aimed at treating the conditions and events resulting from the exacerbation of chronic conditions, in a fragmented, episodic and reactive manner^6^. Such practice becomes an obstacle in the consolidation of the quality of care provided especially in the PHC^7^ ^-^ ^9^.

To fill this gap, the most appropriate health care model to guide the practice is the Chronic Care Model (CCM)^10^. This model guides the provision of chronic care through productive interactions between active and informed users and proactive and prepared health teams. Therefore, the CCM challenges the *status quo* of traditional health programs and emphasizes the importance of rethinking and redesigning the clinical practice at the primary health level. 

One of the instruments that measure the congruence between care measures and the CCM from the perspective of users is the Patient Assessment of Chronic Illness Care (PACIC)^11^, which has been adapted to Brazilian Portuguese^6^. The PACIC emphasizes interactions between users and providers of care, especially aspects of assisted self-care; it includes the users’ evaluation of the frequency with which they are given opportunities to adhere to treatment in the last six months, to understand the care and support model and their participation in decision-making with respect to treatment, setting goals, support for problem solving, and contextualization of counseling, as well as their perception about the coordination of care and follow-up by the local team^6^. This questionnaire has also been considered sensitive to changes in chronic care provision, associated with other measures of productivity and system improvements, mainly with clinical and behavioral outcomes^12^ ^-^ ^14^.

In Brazil, the PACIC has been translated and adapted into Portuguese^6^, since the CCM has been incorporated by the Ministry of Health^15^ into the “Strategic Action Plan to Tackle Noncommunicable Diseases (NCD) in Brazil 2011-2022” as a care model that should subsidize actions directed at the care of people who experience chronic health conditions. 

In this sense, when considering the current movement of reorganization of health services around chronic care, it is still unclear to what extent the assistance to older people with diabetes *mellitus* and/or hypertension in PHC is aligned with the precepts of the Chronic Care Model from the perspective of the elderly themselves, and how this assistance relates to outcomes of the care provided to these individuals.

Despite the discussions undertaken so far, research assessing the quality of care from the perspective of users, especially the elderly, is still limited. Thus, this makes the present study timely. The search for quality of health care services in Brazil has received much attention from the Ministry of health, with particular emphasis on old individuals, taking into account the needs of this age group in the adjustment of public policies. As a matter of fact, the reorientation of health services, with the creation of a culture of chronic care and incorporation of proactive evidence-based care and strategies for improvement of quality, is one of the international recommendations for the Region of the Americas^16^.

In light of the above, and assuming that older people with higher PACIC scores present better care outcomes, the objective of this study was to evaluate the quality of care provided to older people with diabetes *mellitus* and/or hypertension in Primary Health Care, according to the Chronic Care Model, from the perspective of the elderly. We sought specifically to identify associations between the overall PACIC score and the outcomes of the care provided in the sample studied. 

## Method

This cross-sectional study is part of the second phase of the population-based study “Aging and Renal Disease” (en-DoRen), whose overall objective in the first phase was to estimate the prevalence of non-dialytic chronic kidney disease in older people in one of the nine health districts of Belo Horizonte, Minas Gerais, Brazil^17^. The choice of this district was based on the fact that this had the highest absolute number of individuals aged 60 years or older (n = 44,801) at the moment of planning the first phase of the study.

The current analysis was performed with a subsample of the first phase of the en-DoRen study which met the following inclusion criteria: individuals aged 60 years or older; under follow-up of a Family Health team active in the Northwest Sanitary District for at least one year; diagnosed with diabetes *mellitus*, arterial hypertension or both, self-reported or confirmed by the electronic medical record; individual who responded to the PACIC questionnaire. Elderly patients with severe cognitive impairment (Mini Mental State Exam score - MEEM ≤ 9) without a responsible person who could assist in the responses were excluded from the sample.

The en-DoRen study database identified 143 PHC users. Of this total, 118 elderly patients had a medical diagnosis of diabetes *mellitus*, hypertension or both, and were included in the current analysis. There was a loss of 13 people who did not respond to PACIC questionnaire due to death (n = 4), change of address and unsuccessful telephone contact attempt (n = 3), and lack of success to find the person at home after three unsuccessful attempts (n = 6). Therefore, the final sample of this study was composed of 105 older people. 

It is noteworthy that these losses occurred due to the different chronology of approval from the Research Ethics Committee (REC) in the two phases of the en-DoRen study: before the REC approval to start the second phase, 54 elderly had already completed the first phase, and therefore, these elderly had to be approached a second time and invited to respond to the PACIC questionnaire.

The method proposed by Lwanga and Lemeshow^18^ was used for sample calculation, to verify whether the number of participants in the present study was enough to evaluate the quality of care provided by the primary level of health care. To this end, the average prevalence of good quality of care in PHC of 39.7%^19^ ^-^ ^22^, an absolute precision of 10% (the mean standard deviation of the quality of care of the aforementioned studies was 20.4%) and a significance level of 5% were considered, obtaining an estimated sample size of 92 individuals. Taking into account 10% of possible losses, the total estimated number was 101 individuals. Therefore, the sub-sample of this study was adequate to evaluate the quality of care in PHC from the perspective of elderly diabetes *mellitus* and/or hypertension patients.

The data were collected from August 26, 2014, to November 1, 2016, in the homes of the elderly, by two nurses involved in the research and six previously trained undergraduate scholarship fellows. 

Fieldwork involved the application of structured questionnaires and collection of blood and urine samples. Information on sociodemographic, clinical, anthropometric, and biochemical variables, quality of life, and quality of primary care from the perspective of the elderly (PACIC) were collected. 

The PACIC consists of 20 questions distributed into five dimensions: patient activation (3 questions that evaluate the extent to which the individual was motivated and supported by health professionals to initiate changes), delivery system design/decision support (3 questions that assess whether the individual has received support with for example educative material and the extent to which he is satisfied with the care provided), goal setting/tailoring (5 questions that evaluate the extent to which general instructions and suggestions have been adapted to the person’s individual situation), problem solving/contextual (4 questions that refer to how health professionals deal with problems that interfere with the achievement of predefined objectives), follow-up/coordination (5 questions that address how often and how consistently the whole process has been conducted). Individuals can give only one answer to each question whose alternatives are on a 5-point Likert scale, namely: 1) almost never, 2) generally not, 3) sometimes, 4) most of the time, and 5) almost always^6^.

The mean overall PACIC score is obtained by the sum of the scores of each question, divided by the total number of questions (n ​​= 20). In turn, the dimension scores represent the mean scores of the questions in each particular dimension. Higher scores indicate the perception, from the part of users, of greater involvement in self-care and greater support for the care of their chronic conditions^6^.

It should be emphasized that this questionnaire has been adapted and validated semantically and culturally by several groups interested in its use as a support tool for the diagnosis, adjustment, monitoring and evaluation of models of care to chronic conditions grounded in the Chronic Care Model, which has been tested in subjects with various chronic conditions, e.g. diabetes *mellitus* ^11^ ^-^ ^14^ ^,^ ^23^ ^-^ ^24^ ^)^ and cardiovascular diseases^23^ ^,^ ^25^.

Blood pressure and anthropometric data were measured within an interval of up to two weeks after the home visit for application of the questionnaire. On this occasion, the collection of biological material (blood and urine) was scheduled for a maximum period of one week and guidelines were provided for the preparation of the test. The collection of biological material was performed in the morning by two members of the project, after a 12-hour fasting of the patient. The material was sent to a particular clinical laboratory for processing.

The dependent variables in this study were the overall score and the scores of each PACIC dimension. 

The following variables were analyzed: sex (female, male); age in years; level of education in term of complete years of schooling (0-4 and 5 or more); monthly income categorized according to the minimum wage in force in the year of the interview; presence of formal or informal caregiver; polypharmacy (using five or more medications with the presentation of recent medical prescription); smoking (non-smoker, ex-smoker and current smoker); alcoholism measured by the Alcohol Use Disorders Identification Test-Consumption (AUDIT-C) questionnaire^26^ (scores ≥ 4 for males and ≥ 3 for females suggest likely abuse of alcohol); physical activity (practice some sort of physical activity with a frequency of ≥ 3 time/week and for ≥ 30 minutes each session); self-reported morbidities confirmed in the electronic medical record; cognitive level assessed by the MMSE, with cutting point adjusted according to the instructional level of the elderly^27^ (altered cognitive level indicated by scores ≤ 13 in the case of illiterates; scores ≤ 8 years in the case of literates with ≤ 8 years of schooling; scores ≤ 26 in the case of literates with > 8 years of schooling); functional capacity assessed by the Katz index^28^ (scores 0-2: important dependence; 3-4: partial dependence; 5-6: independence); body mass index based on the cut-off points established for the elderly according to literature^29^ (underweight: <22 kg/m^2^; eutrophy: 22-27 kg/m^2^; overweight: 27-30 kg/m^2^ for men and 27-32 kg/m^2^ for women; obesity: >30 kg/m^2^ for men and >32 kg/m^2^ for women); pressure levels categorized later into good control (<140/90 mmHg in hypertensive and <130/80 mmHg in diabetics); self-perception of quality of life (categorized into “good/very good” and “bad/very bad/reasonable”) and satisfaction with the own health (categorized into “satisfied/very satisfied” and “dissatisfied/very dissatisfied/neither satisfied nor dissatisfied”), obtained through the two first questions of the World Health Organization Quality of Life-bref (WHOQOL-bref) questionnaire, version translated and validated for Portuguese^30^. The biochemical variables collected were glycated hemoglobin (HbA1c), total cholesterol and fractions, triglycerides, fasting glucose, albumin/creatinine ratio (ACR), and serum creatinine.

Biochemical parameters were classified within the range of normality and considered in the analysis of care outcomes of elderly people with diabetes *mellitus* and/or hypertension: serum creatinine (<1.3 mg/dL in men and <1.2 mg/dL in women), ACR (<30.0 mg/g), HbA1c (<7% in diabetics and <6.5% in hipertensive), fasting glucose (<126 mg/dL), total cholesterol (<200 mg/dL), high density lipoprotein cholesterol/HDL-c (>40 mg/dL in men and >50 mg/dL in women), low density lipoprotein cholesterol/LDL-c (<160 mg/dL), and triglycerides (<150 mg/dL). Glomerular filtration rate (GFR) was estimated by the Chronic Kidney Disease Epidemiology Collaboration creatinine equation and CKD was defined as GFR < 60 ml/min/1.73 m^2^ and / or presence of albuminuria (ACR ≥ 30 mg/g), confirmed in two laboratory tests with a time interval of ≥ 3 months, according to the criteria for definition of CKD proposed by the Kidney Disease group: Improving Global Outcomes^31^. 

Data were analyzed using the Statistical Package for Social Sciences (SPSS, version 23.0, Chicago, IL, USA). First, the results were analyzed by means of descriptive techniques, expressed in proportions or percentages in the case of the categorical variables, and medians with respective interquartile ranges (IQ) in the case of non-parametric continuous variables. Normality was tested by the Kolmogorov-Smirnov test. The reliability of the PACIC was analyzed using the Cronbach’s alpha coefficient. The Spearman’s-Rho test of the PACIC total score was used to test the correlation between the five dimensions of the questionnaire.

Medians of the overall PACIC scores were compared with care outcomes (biochemical parameters, body mass index, pressure levels and the two WHOQOL-bref questions used) by the U Mann-Whitney test and later adjusted for potential confounding variables (sex, age, schooling, income, time of diagnosis of diabetes *mellitus* and time of diagnosis of arterial hypertension) in the multivariate model through Backward logistic regression. The significance level adopted was 5%, rejecting the null hypotheses of the absence of differences when the p-value was less than 0.05. The values ​​obtained were expressed in *odds ratio* (OR) and their respective 95% confidence intervals (95% CI).

The study was approved by the Research Ethics Committee of the Federal University of Minas Gerais (Opinion nº 1,238,099) and by the Municipal Health Department of Belo Horizonte (Opinion nº 1,351,378), observing all legal procedures. Participants were informed about the objectives of the study and signed the Informed Consent term, guaranteeing confidentiality and anonymity. 

## Results

The sample consisted of 105 elderly individuals, with a predominance of females (67.6%). The age ranged from 60 to 93 years, with a median of 72.0 years (IQ 66.5-80.5 years). Low schooling was predominant among the participants (56.2% reported 0 to 4 complete years of schooling). Just over ¼ of the sample reported a monthly income of ≤ 1 minimum wage. The presence of a caregiver was reported by 11.4% of the sample. Regarding behavioral habits, 10.5% of the elderly were smokers, 19.0% had a probable diagnosis of alcohol abuse, and only 20.0% practiced some type of physical activity. 

The majority of the elderly had a good or very good perception of quality of life (65.0%) and was satisfied or very satisfied with their health (57.3%). Altered cognitive levels were present in 10.5% of the elderly, and two of them presented a score ≤ 9 points in the MMSE and were under the care of a caregiver. Regarding activities of daily living, only 1.9% of the elderly had partial or total dependence. There was a high prevalence of dyslipidemia (86.7%), CKD (30.5%) and heart disease (20.0%), and 13.3% reported previous cerebrovascular accident. Only 28.6% of the elderly were eutrophic and 21.9% were obese. Among the hypertensive elderly (n = 104), 42.9% had a concomitant diagnosis of diabetes *mellitus*. The median duration of hypertension was 13 years (IQ 7.0-23.0 years) and of diabetes *mellitus* was 7.0 years (IQ 4.0-13.0 years). Polypharmacy was present in 65.7% of the elderly.

Metabolic control and blood pressure levels were adequate in 74.3% and 54.3% of the elderly, respectively. Among the biochemical parameters investigated, the worst indicator was HDL_c (50.5%), followed by triglycerides (65.0%) and total cholesterol (71.0%). Detailed information on care outcomes in the sample studied is presented in Table 1.

The reliability of the PACIC questionnaire was satisfactory according to the Cronbach’s alpha coefficient (0.881). There was also a moderate to strong correlation between the five PACIC dimensions and the overall PACIC score, ranging from 0.490 (patient activation) to 0.889 (goal setting/tailoring), all statistically significant (p <0.001). 

**Figure 1:**
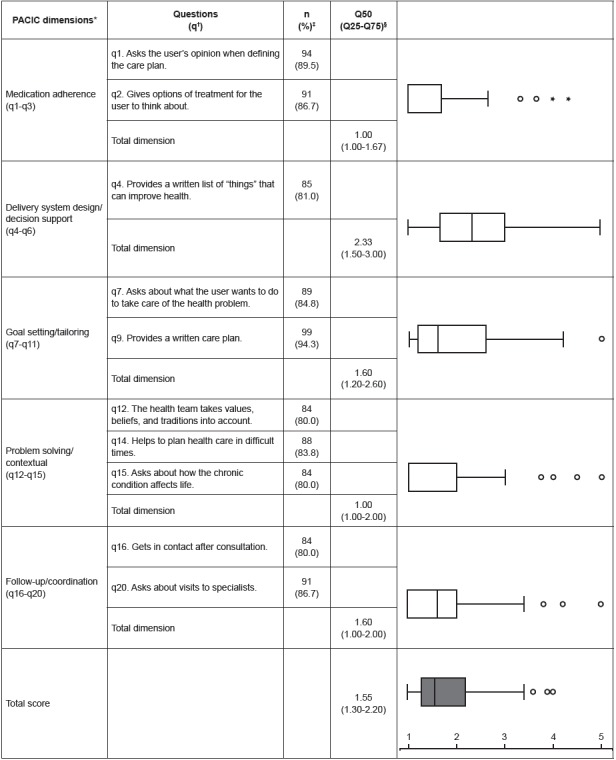
Descriptive distribution of the 10 items with the worst evaluation and the overall score and boxplots of the five dimensions of the Patient Assessment of Chronic Illness Care. Belo Horizonte, MG, Brazil, 2014-2016

Note: Likert-type scale from 1 to 5 (1 = lowest agreement, 5 = highest congruence with the Chronic Care Model - CCM). It was decided to highlight the 10 questions with the lowest evaluation among the 20 evaluated items. * PACIC - Patient Assessment of Chronic Illness Care; † q - question; ‡ Percentage of people who answered “almost never”; § Q - quartile.

The elderly attributed a low median score to the quality of care received according to the overall PACIC score (1.55; IQ 1.30-2.20). As for dimensions, it was observed that the delivery system design/decision support presented a better result (2.33, IQ 1.50-3.00), while Patient activation (1.00, IQ 1.00-1.67), Problem solving/contextual (1.00, IQ 1.00-2.00) and Follow-up/coordination (1.60; IQ 1.00-2.00) stood out as frailties from the perspective of the elderly (Figure 1). 

**Table 1:** Care outcomes among older people with diabetes *mellitus* and/or hypertension followed-up in the Primary Health Care system. Belo Horizonte, MG, Brazil, 2014-2016

Outcomes measures		
Perception of overall quality of life*	n	%
Good/very good	67	65.0
Neither bad nor good/bad/very bad	36	35.0
Satisfaction with health*		
Satisfied/very satisfied	59	57.3
Neither satisfied nor dissatisfied/dissatisfied/very dissatisfied	44	42.7
Body mass index (kg/m^2^)		
<22	11	10.5
22-27	30	28.6
27-30 (Males) and 27-32 (Females)	41	39.0
> 30 (Males) and > 32 (Females)	23	21.9
Pressure levels	Median	Q25 - Q75†
Systolic blood pressure (mmHg)	130.00	120.00 - 140.00
Diastolic blood pressure (mmHg)	74.00	70.00 - 80.00
Biochemical parameters		
Fasting glucose (mg/dL)	100.00	88.00 - 115.0
HbA1c^‡^ (%)	6.10	5.65 - 6.80
Total Cholesterol (mg/dL)^§^	182.50	164.00 - 206.75
LDL-c|| (mg/dL)^¶^	106.00	87.00 - 128.00
HDL_c** (mg/dL)^¶^	45.00	40.00 - 54.00
Triglycerides (mg/dL)^§^	126.50	97.25 - 171.75
Albumin/creatinine ratio (mg/g)	8.05	3.73 - 15.98
Serum creatinine (mg/dL)	0.86	0.70 - 1.01
Glomerular filtration rate (mL/min/1.73 m^2^)^††^	74.76	61.55 - 87.21

Note: * Two cases of missing information; † Q - quartile; ‡ HbA1c - glycated hemoglobin; § Five cases of missing information; || LDL_c - low density lipoprotein cholesterol; ¶ Six cases of missing information; ** HDL_c - high density lipoprotein cholesterol; †† Glomerular filtration rate estimated by the Chronic Kidney Disease Epidemiology Collaboration creatinine equation.

**Figure 2:**
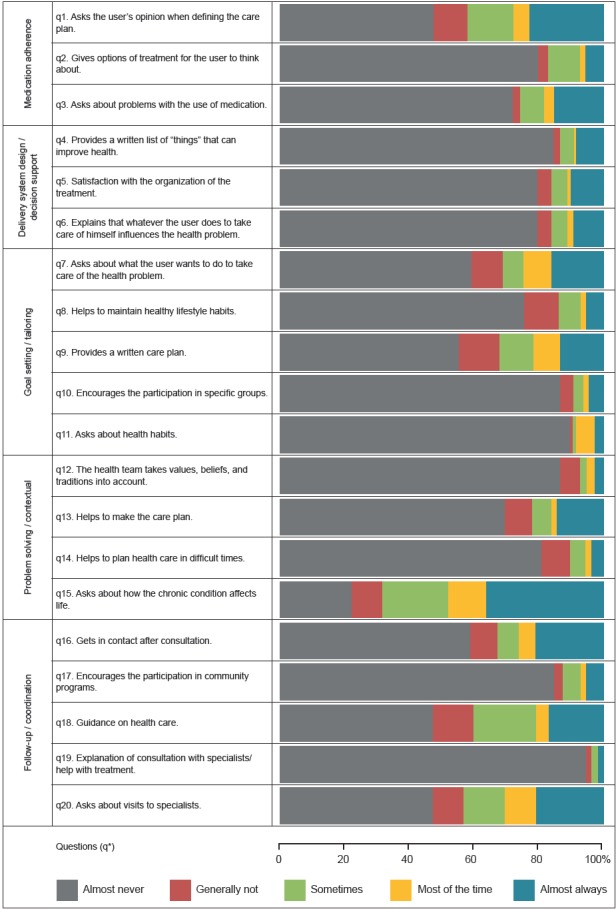
Descriptive distribution of the five dimensions of the Patient Assessment of Chronic Illness Care (PACIC) for each question. Belo Horizonte, MG, Brazil, 2014-2016

Note: * q - question.

Regarding the answers to each item in the questionnaire, it was observed that more than half of the elderly mentioned “almost never” in response to 16 out of the 20 evaluated items, where the items 1 and 9 had the highest concentration of respondents (89.5% and 94.3%). Only item 5 presented a predominance of the “almost always” response (36.2%) (Figure 2).

**Figure 3:**
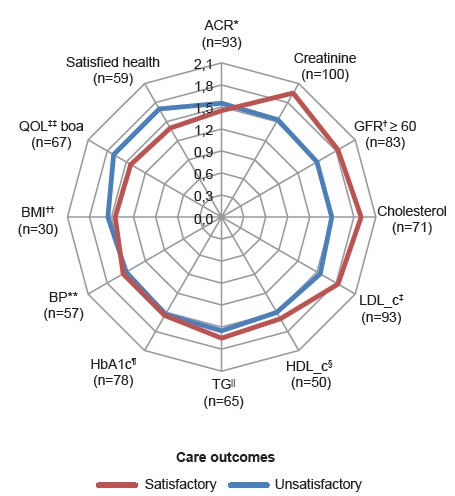
Median of the total scores of the Patient Assessment of Chronic Illness Care (PACIC) according to satisfactory or unsatisfactory care outcomes. Belo Horizonte, MG, Brazil, 2014-2016

Note: * ACR - Albumin/creatinine ratio; † GFR - glomerular filtration rate; ‡ LDL_c - low density lipoprotein cholesterol; § HDL_c - high density lipoprotein cholesterol; || TG - triglycerides; ¶ - HbA1c - glycated hemoglobin; ** BP - blood pressure; †† BMI - body mass index; ‡‡ QOL - quality of life. n = number of elderly people with satisfactory care outcome. Controlled HbA1c: <7% (diabetic) and <6.5% (hypertensive). Controlled BP: <140/90 mmHg (hypertensive) and <130/80 mmHg (diabetics). p>0.05 adjusted for sex, age, schooling, income, time of diagnosis of diabetes *mellitus* and time of diagnosis of hypertension.

No statistical difference was observed between the medians of the overall PACIC score and individual care outcome indicators (Figure 3), even after adjusting for sex, age, schooling, income, time of diagnosis of diabetes *mellitus* and time of diagnosis of hypertension. However, when the median differences in overall PACIC scores were evaluated among the elderly who reported good or very good quality of life and those who were satisfied or very satisfied with their health, a statistically significant difference was found between medians (1.83 vs. 1.40, p = 0.019), adjusted for the aforementioned variables. These elderly people were 2.01 times more likely to have higher total median PACIC scores than the other elderly patients (OR = 2.01, 95% CI: 1.12-3.59, p = 0.019) (data not shown).

## Discussion

In this study, the overall PACIC score of 1.55 indicates that, in general, the congruence between the assessed care process and the CCM never occurred or occurred few times from the perspective of the participants. This result contrasts with other international studies that reached a total score higher than that presented in the current research, ranging from 2.33 to 4.19^12^ ^-^ ^14^ ^,^ ^23^ ^-^ ^25^ ^,^ ^32^. The only study at the national level, the one responsible for the translation of the questionnaire in the country, in Curitiba, reported a mean score of 2.86^6^. Part of this difference can be explained by the fact that the cited studies included younger people with other morbidities. Another relevant aspect is the possible influence of the presence of greater cultural homogeneity in other countries when compared to the Brazilian reality.

The low score found suggests weaknesses of Family Health teams in ensuring proactive, planned, coordinated and patient-centered care^6^. These flaws are reflected in possible difficulties in incorporating non-clinical aspects of chronic care into the practice, as for example, the implementation of assisted self-care^16^. 

This is one of the key elements of the CCM to ensure a high quality of care. The Pan-American Health Organization considers a fundamental and innovative strategy to assist people with chronic health problems. Once the chronic condition is diagnosed, either diabetes *mellitus* or hypertension, the patient will need to deal with this condition in the daily life and, consequently, self-care will be a life-long task for the patient and his family^16^. To emphasize the importance of self-care, we stress that people with diabetes *mellitus* spend about 8.7 hours per year with a health professional, and during the other 8,751.3 hours, they manage the illness by themselves. Therefore, it is imperative to help these individuals understand and take on the responsibility for their illness^33^. 

By definition, assisted self-care consists of the systematic implementation of education and support interventions by health professionals in order to increase the users’ ability and confidence in managing their health problems, promoting a sense of co-responsibility. It includes the choice of problems to be prioritized, fixation and monitoring of the fulfillment of goals focused on the needs, values​, and preferences of the user, identifying the difficulties to fulfill them, support for the solution of problems based on a care plan constructed with the user, among other actions. This way, care providers are no longer prescribers but become partners of people who use health care systems^16^.

Based on the above, questions about the training of health professionals are raised, as this may not give proper emphasis to a care approach that recognizes the crucial role of users in managing their own health condition. This questioning is shared by other authors^24^ ^)^ and it is supported in the literature. A research developed in Quebec with 364 diabetes *mellitus*, hypertension and chronic obstructive pulmonary disease patients (mean age: 64.9 ± 11.8 years), users of educational institutions, found on the one hand a mean overall PACIC score of 2.8 and, on the other hand, a high quality of the technical care with almost 80% of adherence to the clinical guidelines for the chronic conditions studied. The authors explained that this fact may have occurred in part by the academic context itself, whose clinical teaching, that it is focused on training, may direct less attention on the implementation of actions aligned with the CCM’s benchmarks than on technical quality, which is more easily evaluated^23^. 

However, the low PACIC scores may also indicate that, even if these actions have been implemented in practice, they are not adding value to people, because they were not recognized by users. This is because although the term self-care is well inserted in the discourse of the health education field, its exact understanding and authentic application do not always occur so easily because it often implies a paradigm shift. Complementing what has already been commented, traditional biomedical health care models directed to acute and episodic conditions often support the formation and socialization of health professionals, so that when these professionals try to incorporate their principles into their current beliefs and practices, many misconceptions about the subject arise. Thus, professionals need to become aware of such contradictions and undertake changes of attitude or philosophy^34^.

In turn, when analyzing each individual PACIC item, it was found that only four questions obtained medians above 1.00 (q5, q8, q10, and q11). However, only q5 “Were you satisfied with the organization of your treatment” obtained a score above the average of the total possible score to be scored, with a predominance of the “almost always” response (36.2%), with the proviso that 21.9% of the elderly said they were “almost never” satisfied. The contrast between the low scores of the other questions with greater satisfaction with the health service should be interpreted with caution, since it is possible that the elderly in this study have higher expectations regarding the health care received and this, consequently, influences the perception of the quality of care offered by the Family Health teams. Future research should explore in greater depth the relationship between quality of care and satisfaction in this population segment. 

Among questions with high percentages of “almost never” answers, we highlight q1 (Asks the user’s opinion when defining the care plan - 89.5%), q2 (Gives options of treatment for the user to think about - 86.7% %), q7 (Asks about what the user wants to do to take care of the health problem - 84.8%) and q12 (The health team takes values, beliefs and traditions into account when indicates the treatment - 80.0%). This finding is worrisome since it evidences possible failure of health professionals in recognizing the responsibility of users to make decisions about their own care. In this perspective, the lack of flexibility in care choices may lead to the idea, although implicitly, that the individual will have his life controlled by the disease. This situation in the context of elderly people with diabetes *mellitus* and/or hypertension can lead to unfavorable clinical and functional outcomes, insofar as the metabolic consequences are a function of the decisions and actions that people make during the daily management of the illness^34^ ^-^ ^35^.

This is because the discovery of a chronic condition requires that people change their daily life so as to be able to organize the care, from the development of skills to handle a range of activities whether or not predicted by medical knowledge, including adverse conditions for the control of the disease imposed by the socio-cultural context in which the patient is inserted, a situation called by some authors as “rupture of the biography of the individual”. However, over time^36^, patients may develop an attitude of “strategic lack of adherence”, unconsciously and critically failing to comply with medical recommendations^36^. In this sense, some strategies aimed at supporting effective self-care can be used by Family Health teams. They are, for example, “behavioral strategies” (empowerment - discovery and development of the individuals’ capacity, valuing their autonomy and responsibility for their own life; “support groups”, “problem solving”; “motivation and support for autonomy”)^36^. 

Regarding the factors associated with the PACIC, the results did not support the hypothesis that older people with higher PACIC scores present better care outcomes. However, in the joint analysis of the questions related to the perception of quality of life and satisfaction with health, the hypothesis was upheld. These findings indicate the need to re-signify the practice beyond technical and laboratory care to maintain high levels of quality of care.

The findings demonstrate that it is important to highlight the important role of Nursing in PHC as a driving force for change. Its essence, the “care”, provides spaces of intersubjective encounter between professionals and the persons who experience chronic health conditions so necessary for the development of attitudes/behavioral changes. It is a slow and difficult process for people with chronic conditions, as it involves rethinking the whole routine and adapting the life project. In this sense, the performance of nurses has great potential to act according to the precepts of chronic care, be it in the nursing consultation, or in individual or collective educational activities, and even in mobilization actions in the community. This can be achieved through a critical-reflexive assimilation of knowledge that makes it possible to arise awareness of the new health condition in an autonomous way. In this context, Nursing is able to rescue the intersubjectivity, involving reflection and action, allowing to the others to problematize their situation. Freedom starts filling the space previously inhabited by the persons’ dependence and in this way they discover how to participate in the transformation of their world towards the integral health of the human being^37^.

As limitations of the study, we highlight the cross-sectional nature of the study that makes it impossible to determine causal relationships of the outcome and variables of interest. There were also no national or international studies that included only elderly people using public health services for comparison purposes, and the bias of selective response cannot be excluded. As potentialities of the study, we highlight a population-based and randomized sample among the census sectors; the use of a questionnaire that has been translated and adapted to Portuguese, contributing to the accuracy of the answers given in the assessment of the quality of care; and the absence of *missing* cases in the PACIC questionnaire, which the literature indicates that can reach up to 32.7%^25^ ^,^ ^32^. 

## Conclusion

Poor quality of care provided for older people with diabetes *mellitus* and/or hypertension was found in the Primary Health Care according to the Chronic Care Model from the perspective of the elderly. This indicates that the reorganization of the care model oriented towards chronic care in the context of PHC seems still to fall short of its precepts, giving way to traditional biomedical models, from the perspective of the study participants. 

It was not possible to confirm the hypothesis that older people with higher PACIC scores present better care outcomes, only in the case of older people who reported good or very good quality of life and who were simultaneously satisfied or very satisfied with health. 

We recommend the expansion of strategies *in loco* that make it possible the diversification of prevention and management actions of health conditions that include the culture, values, and experiences of users. 
